# Development of the Kisiizi hospital health insurance scheme: lessons learned and implications for universal health coverage

**DOI:** 10.1186/s12913-018-3266-8

**Published:** 2018-06-15

**Authors:** Sebastian Olikira Baine, Alex Kakama, Moses Mugume

**Affiliations:** 1 Department of Health Policy, Planning and Management, Makerere University College of Health Sciences, School of Public Health, P. O. Box 7072, Kampala, Uganda; 2 Kisiizi Hospital Health Insurance Scheme, Kisiizi Hospital, P. O. Box 109, Kabale, Uganda

**Keywords:** E-society, Solidarity, Insurance, Financial protection, Access, Quality health services

## Abstract

**Background:**

Kisiizi Hospital Health Insurance scheme started in 1996 to; improve access to health services, and provide a stable source of funding and reduce bad debts to Kisiizi hospital. Objectives of this study were; to describe Kisiizi Hospital Health Insurance scheme and to document lessons learned and implications for universal health coverage.

**Methods:**

This was a descriptive cross-sectional study. Data from different sources were triangulated and thematically analysed.

**Results:**

Most households (96%) were organized in Engozi societies (e-Societies), met monthly, and made financial contributions. Cultural solidarity in e-Societies provided a platform for the Kisiizi hospital health insurance scheme establishment, operation and made it compulsory for members. e-Societies disciplinary measures and fear of high out-of-pocket payment for health care enforced enrolment, retention and increased membership. Community sensitisation and community participation in setting premiums and co-payments provided for better understanding of health insurance and rendered them acceptable, affordable and equitable. Membership increased from 330 in 1996 to 38,400 families in 2017. Kisiizi hospital health insurance scheme covered only health services obtained from Kisiizi hospital. Kisiizi hospital health insurance scheme offered no exemption, credit and referral facilities. e-Societies sometimes paid premiums for members from savings and offered them loans to. Kisiizi hospital provided good quality health services, which were easily accessed by insured members. Kisiizi hospital got a stable source of funding and reduced debt burden.

**Conclusions:**

Kisiizi hospital health insurance scheme improved access to health services, provided a stable source of funding and reduced bad debts to the hospital. Internal and external factors to e-Society enforced enrolment and retention of members in Kisiizi hospital health insurance scheme. Good quality health services at Kisiizi hospital demonstrated value for money and offered incentives for enrolment and retention, and coverage expansion. Community sensitization and participation in setting premiums and co-payments rendered Kisiizi hospital health insurance scheme acceptable, affordable and catered for equity. Insured members enjoyed benefits; protection against catastrophic health spending, impoverishment, and easy access to quality health care.

**Electronic supplementary material:**

The online version of this article (10.1186/s12913-018-3266-8) contains supplementary material, which is available to authorized users.

## Background

Health insurance has been perceived to enhance access and utilization of good quality health services, and to provide protection against catastrophic health expenditure [1]. Evidence shows that health insurance improves utilization of and accessibility to health services [2, 3], and diminishes catastrophic health expenditure among insured households [4].

Several countries in Sub Saharan Africa have implemented community-based health insurance schemes [5] in which communities are actively engaged in resource mobilization and management [6–8]. Evidence shows that availability of essential drug improved at health facilities operating community-based health insurance schemes [9].

However, community-based health insurance schemes are characterized by: low coverage; low premiums; lack of trust; poor understanding of insurance; poor quality health services, high operational costs and limited health benefit packages [2, 7, 9, 10]. These issues render community-based health insurance scheme financially unsustainable and reliant on external support.

Kisiizi Hospital Health Insurance scheme is a community-based health insurance scheme owned by Kisiizi hospital, a faith based hospital in South Western Uganda. At the time of the launch (1996), patients sought health care at the Kisiizi hospital when illnesses were in advanced stages. Delays to seek care were mainly due to lack of ready and adequate cash to spend on health care [11, 12]. This most often led to long hospital stays or treatments associated with higher health care bills and sale of household assets (especially land and domestic animals) that in turn led them to impoverishment. Some patients escaped from the hospital beds before complete recovery without settling their health care bills. By the end of 1994, unpaid debts by patients accounted for about 2.5% of the total annual recurrent costs of Kisiizi hospital [13].

The pressure of maintaining good quality health services had compelled the hospital to raise the prices of care to levels beyond which further increase would significantly impede health service utilization by residents in the hospital catchment area. The objectives of Kisiizi Hospital Health Insurance scheme establishment were three fold: to improve access to health services; to provide a stable source of funding for the hospital; and to reduce the problem of bad debts.

Most households in villages in the Kisiizi hospital catchment area were organised in Engozi societies (e-Societies). Engozi is a locally made stretcher used to transport patients to hospital and at times to bring back home the dead loved ones for descent burial. A household with a severely sick person sounded a drum in a particular rhythm to invite e-Society members to carry the patient in turns to hospital [14].

e-Society tradition brings people together to address a common problem of rugged land terrain that is unfavourable for motor vehicle ambulance use. In addition, some patients cannot afford the cost of transport by ambulance and resort to this local means.

e-Society members were eligible to register in Kisiizi Hospital Health Insurance scheme provided they paid the required premiums and co-payments. Photographs and membership information of all registered members were entered into a computer based system. Eligible members were able to access services two weeks after effecting payments. Premium payment receipts work as membership cards to all family members. The objectives of this study were; to describe Kisiizi Hospital Health Insurance scheme, and to document lessons learned and policy implications.

## Methods

This was a descriptive cross-sectional study where both quantitative and qualitative methods were applied. Three principle investigators collected data in July 2017 through: a review of Kisiizi Hospital Health Insurance Scheme documents to access documented evidence; key informant interviews with three health providers and three chairpersons of e-societies with the aim of collecting information from stakeholders with firsthand knowledge about Kisiizi Hospital Insurance Scheme and its operations using key informant interview guide (Additional file 1); and one group discussion with twenty community representatives (chairpersons of e-societies) and three focus group discussions each comprising of eight randomly selected insured members from the community were done with the aims of generating data and insights on Kisiizi Hospital Insurance Scheme from memories, ideas, experiences, perceptions, opinions, and attitudes of stakeholders in an interactive group setting with freedom to talk among group members using a guide (Additional file 2). Transcription and translation were verbatim by Principal Investigators who are fluent in both English and local languages. Data from these sources were triangulated, coded, and themes were generated. A thematic analysis then followed.

## Limitations

This study had study limitations that included:

1. The study did not include non-insured households and a comparative analysis of the experiences of poor and non-poor insured cannot be provided to enable assessment of the extent to which the poor people have affordable access to quality health care.
2. Limited funding and time did not allow conducting a more complex study to include auditing financial transactions, renewals and health service utilisation. These will be done in the planned study.
3. The study was qualitative and missed out valuable quantitative inputs.

## Results

### Engozi society

Majority of households (96%) in the Kisiizi hospital catchment area were members of e-Societies. They met once every month and made financial and other forms of contributions. Requirements to enrol into Kisiizi Hospital Health Insurance scheme were being a member of e-society, and payment of premium and copayment. Renewal is usually enforced by e-society demands. The hospital finance office adds up the costs of services consumed by members monthly and gives the total expenditure by members to the scheme to reimburse.

e-society savings are available for loaning to members to generate income. Sometimes, e-Societies paid premiums for members in Kisiizi Hospital Health Insurance scheme using savings. Solidarity that exists in e-societies provided a platform for implementation and operation of Kisiizi Hospital Health Insurance scheme. Participants in focus group discussions concurred that;

“e-Societies reserved funds were made available for borrowing in case of failure to raise money needed to pay premiums and co-payments” [Focus Group Discussions 1,2,3].

Families who were not members of e-Societies and/or members of Kisiizi Hospital Health Insurance scheme faced severe disciplinary actions when in need of support from e-Societies members. Disciplinary measures included; refusal to transport the patient to the hospital, refusal to transport the dead back home in case the patient died while at the hospital, and refusal to participate in the burial process until a penalty was settled. Penalties were in form of cash or slaughtering a goat of their choice and preparing a banquet for them to feast at the family cost.

### Membership into the Kisiizi hospital health insurance scheme

Membership in the Kisiizi Hospital Health Insurance scheme gradually increased over 21 years as shown in Table 1.

**Table 1:** Membership in the Kisiizi Hospital Health Insurance scheme

Year	Number of households registered in KHI scheme
1996	330
1999	1172
2017	38,400

Sources: Kisiizi Hospital Health Insurance reports 1996, 1999 and 2017

Increase in membership was attributed to: continuous sensitization of the communities and increased understanding of the insurance concept and benefits; community participation; fear of e-Society disciplinary measures and high costs of health care; and increased geographical coverage from three sub-counties to seven districts or up to a radius of 60 km from the hospital. Despite the increase in membership, over 235,334 households in the catchment area are still not insured.

Kisiizi Hospital Health Insurance scheme was understood as a financing method where households put efforts together and shared the burden of diseases in order to reduce costs of health services to individuals. Participants acknowledged that;

“The burden of large sums of money required before the Kisiizi Hospital Health Insurance scheme was no longer a hindrance to insured households. Raising premiums and co-payments was not as strenuous as raising funds to pay full health care costs” [Focus Group Discussion 3].

"My brother has been to the hospital three times and he has not sold portions of his land to pay for his health care because of being a member of Kisiizi Hospital Health Insurance scheme" [key informant 1].

e-Society leaders registered family members, collected premiums, and submitted a lump sum to the Kisiizi Hospital Health Insurance scheme office. They were given family receipts, which worked as membership cards to all family members.

### Premiums and co-payments

Premiums depended on household size while co-payments are hinged on whether care was provided on an outpatient or inpatient basis as shown in Tables 2 and 3 respectively.

**Table 2:** Annual premiums

Number of people in a household	Premium/per person/annum (United States Dollars)
1	7.79
2–4	3.89
5–8	3.06
9–12	2.78
Above 12	4.17 per extra person

Source: Kisiizi Hospital Health Insurance report, 2017

**Table 3:** Co-payments

Policy	Co-payment per visit (United States Dollars)
Outpatient services	0.56
In patient services:	
Children’s ward	2.78
Medical condition	5.56
Normal delivery	5.56
Surgery	16.69

Source: Kisiizi Hospital Health Insurance report, 2017

Average exchange rate: 1 US dollar to 3595.90 Uganda shillings (Source Bank of Uganda June 30, 2017).

Co-payment covered examination by a clinician, common laboratory investigations and medicines.

Kisiizi Hospital Health Insurance did not have specific benefit packages. The premium and copayments catered for any type of health services received except for the excluded services.

Kisiizi Hospital Health Insurance scheme paid for healthcare services obtained from Kisiizi Hospital only. Kisiizi Hospital Health Insurance scheme did not cover cost of care obtained from other sources including referred patients. Kisiizi Hospital Health Insurance scheme paid up to US$ 556.2 per person per episode of sickness and remained with a net balance. Kisiizi Hospital Health Insurance scheme had no external donations or did not need to borrow money, and had no patient debts to the hospital.

### Sources of household income

Sources of household income used to pay premiums and co-payments were sale of agricultural products, small retail shops, brick making, wood products and sell of manual labour. Participants in all focus group discussions indicated that all households were able to pay the premiums and co-payments charged Kisiizi Hospital Health Insurance scheme.

“Every household was capable of raising funds needed to pay premiums and co-payment required in Kisiizi Hospital Health Insurance scheme. Even the poor who were willing to enroll into the scheme generated required funds generated from sell of manual labour” [Focus Group Discussions 1,2,3].

All participants revealed that households needed little money for co-payment compared to large sums of money required to obtain health services at Kisiizi hospital when not insured. In one focus group, participants said;

“Premiums and co-payments in Kisiizi Hospital Health Insurance scheme were fair and affordable compared to the large sums of money required if not a member of Kisiizi Hospital Health Insurance scheme” [Focus Group Discussion 1].

“Sell of manual labour was a more reliable source of income”. The consensus was that every household was capable of raising resources needed to pay for health services provided. The barrier was that households shun the sell of manual labour because of the perceived social stigmatization.

### Services covered by the Kisiizi hospital health insurance scheme

Kisiizi hospital provided a wide range of good quality health services for medical and surgical conditions, casualty and emergency services, out-patient services, in- patient services, special investigations including X-ray, Ultrasound, ECG (heart tracing), and laboratory investigations. Key informants from the hospital attributed good quality services to the presence of adequate trained health providers, support staff, logistics and medical supplies. They are all coverage by the Kisiizi Hospital Health Insurance scheme with some exceptions as detailed in the following sections.

Services that were covered 100% by Kisiizi Hospital Health Insurance scheme included: outpatient and inpatient medical and surgical conditions; emergency services; investigative procedures such as X-ray, ultrasound, electrocardiogram and laboratory investigations; and in-patient services due to complications of chronic diseases provided evidence of good adherence to treatment guidelines was shown.

Services covered 50% by the Kisiizi Hospital Health Insurance scheme were mainly elective surgery such as; prostatectomy, hysterectomy, removal of fibroids, tubal ligation, mastectomy and surgery of the goitre.

### Services that were excluded in Kisiizi hospital health insurance scheme

Services not covered by Kisiizi Hospital Health Insurance scheme included: dentures; spectacles/optical appliances; self-inflicted injuries such as complications of failed suicide attempt, criminal abortion and fights under influence of alcohol and other substance abuse; chronic diseases such as diabetes, high blood pressure, asthma, epilepsy and other mental illnesses; fertility treatment and procedures; food and food supplements; police forms; routine medical checkup for jobs or school; semen analysis; culture and sensitivity; glycated haemoglobin test; international normalized ratio assays evaluating the extrinsic pathway of coagulation; hormonal tests (prostate surface antigen test and thyroid-stimulating hormone blood test); patient referrals and care obtained from outside Kisiizi hospital.

### Exemption and credit facilities

There were no exemption and credit facilities for the poor households in Kisiizi Hospital Health Insurance scheme. Inequities were addressed through community participation in setting premiums and co-payments, and in the management of Kisiizi Hospital Health Insurance scheme. e-Society chairpersons and elected executive committee of community representatives on the Kisiizi Hospital Health Insurance scheme management conceded that;

“All households were potentially able to pay premiums and co-payments. Inequities did not arise in Kisiizi Hospital Health Insurance scheme” [e-Society Chairpersons and Community representatives].

Participants revealed that non-insured household members were simply not willing to pay premiums. They said non-insured households argued that:

“premiums were not transferrable across contract periods in case no family member fell sick in one contract period; Kisiizi Hospital Health Insurance scheme did not cater for health care obtained from other sources; and wanted to invest the money for premiums into business and make out-of-pocket payment using interest generated by the trade” [e- Society Chairpersons and Community representatives].

### Challenges

Major problems faced were mobilising e-Society member families to subscribe Kisiizi Hospital Health Insurance scheme, bad past experience with insurance companies which never honoured their obligations, and households had not explicitly understood the importance insurance, how it operates and the benefits. Also the belief that households paid taxes and government had a free health care policy discouraged and delayed enrolment and retention in Kisiizi Hospital Health Insurance scheme.

## Discussion

Households lacked financial risk protection mechanisms and experienced a high burden of health expenditure, escaped from hospital beds before complete recovery without settling bills, sold assets, and owed the hospital unpaid debts. This was similar to what was observed especially in rural settings in other countries [15].

e-Societies were formed to respond to common problems; difficult transport due to rugged terrain and challenges during burials. Members of e-Societies worked toward measurable goals; they pooled resources and shared health risks. There were unwritten guidelines and code of conduct to ensure a smooth running of e-Societies business; members met monthly on a specified agreed day, made financial and other forms of contributions as need arose. Benefits outweighed transactional costs [16]. This was characteristic of effective teams when players come together to achieve common goals [17–19].

Cultural solidarity in e-Societies was an important tool in the establishment of Kisiizi Hospital Health Insurance scheme while qualities of e-Societies enforced enrolment, retention of membership. Cultural solidarity in e-Societies and their qualities have led to the establishment of many similar health insurance schemes e.g. Ishaka Hospital Insurance scheme, Nyakibale Hospital Health Plan, Kitanga Health insurance scheme, Nyamwegabira Health Insurance scheme [20]. Enrolment and retention in Kisiizi Hospital Health Insurance scheme was not solely attributable to e-Society dynamics alone. There were also external factors to e-Societies that strengthened enrolment and retention of members in Kisiizi Hospital Health Insurance scheme such as fear of high costs of health care, fear to sell household assets to pay for health care, and the desire to access the good quality health services available at Kisiizi hospital without incurring catastrophic health expenditure. This is characteristic of society [19].

Kisiizi hospital should not risk exploiting the solidarity in the e-Societies in order to generate funds for the hospital. This would weaken Kisiizi Hospital Health Insurance scheme’s credibility and even lead it to collapse when it lose trust and attraction among others as evidence suggested [21].

Kisiizi Hospital Health Insurance scheme membership growth was a composite product of community sensitization, increased understanding insurance, appreciation of benefits, e-Society enforcement, sharing of experience between insured and uninsured households, affordable sources of funds for households, and desire to continue accessing quality care at a low cost. Every household could afford to sell non-traditional cash crops and manual labour. In addition, e-Society had a loan facility from which members could acquire credit at affordable interest rate similar to savings and credit schemes [22, 23]. From time to time, e-Societies paid premiums for members using savings and this relieved Kisiizi Hospital Health Insurance scheme members that lacked reserved funds from the stress of raising funds from other sources.

Strong community participation was a strength in Kisiizi Hospital Health Insurance scheme. Chairpersons of e-Societies registered households for Kisiizi Hospital Health Insurance scheme, collected premiums from members, and delivered collections to the Kisiizi Hospital Health Insurance scheme office. Elected representatives of the community participated in setting premiums, co-payments and management of Kisiizi Hospital Health Insurance scheme. Community participation enhanced acceptance, enrolment, and retention that in turn increased Kisiizi Hospital Health Insurance scheme membership and generation of funds. Community participation also offered an opportunity to match premiums and co-payments to existing household resources, address house income disparities, reduce inequities and increase access to care as evidence suggested [24, 25].

Although cultural solidarity in e-Societies and Kisiizi Hospital Health Insurance scheme offered an opportunity to tap into household resources, inequities still existed and were a challenge because household had different income levels. Evidence showed the incidence of catastrophic health spending was high in rural settings and out of pocket payments were even higher [19]. Further evidence available suggested that Kisiizi Hospital Health Insurance scheme may not achieve both increase access to health services and reduce inequities simultaneously [26]. Evidence suggested a well-functioning primary health care network as an appropriate intervention mechanism to increase equity in access to quality health services [27, 28].

Success factors for Kisiizi Hospital Health Insurance scheme were cultural solidarity and enforcement by e-Societies disciplinary measures, resilience, continuous community sensitization and participation in all aspects of Kisiizi Hospital Health Insurance scheme, fear of high costs of health care, and incentive generated availability of good quality health services at Kisiizi hospital. The other success factors included community understanding of insurance and appreciation of benefits of being members of the Kisiizi Hospital Health Insurance scheme; cross learning between insured and non-insured members; protection against catastrophic health expenditure, had peace of mind with no much worries about medical bills or selling household assets, and access to good quality health services.

It is still early for Kisiizi Hospital Health Insurance scheme to celebrate any wins. Currently there is no health financing mechanism is an end to itself. Kisiizi Hospital Health Insurance scheme generated additional funding for health but other sources of funding were still needed to buffer the decreased funding especially when membership drops [29]. There was uncertainty in revenue generation because households depended heavily on agriculture for income and harvests. Like Tanzania, serious drought has been experienced which led to repeatedly poor harvests. This could lead to a fall in enrolment, retention and membership [30, 31]. Evidence available suggests that community based health insurance schemes are not yet stable [32]. This is an issue for Kisiizi hospital to consider and prioritize and develop sustainability strategies for Kisiizi Hospital Health Insurance scheme.

A significant proportion of households were not insured. A bad past experience with insurance companies, which never honoured their obligations made it difficult to mobilise community members to subscribe to Kisiizi Hospital Health Insurance scheme. Others did not understand the importance of health insurance, how it operated and benefits. They did not perceive value for money in Kisiizi Hospital Health Insurance scheme and instead argued they could invest the money in business and pay cash (out of pocket) for health care from interest.

Development process of Kisiizi Hospital Health Insurance scheme to date had lasted 21 years but positive progress has been registered. This was a similar experience to what established insurance schemes went through [33]. Development of any health insurance scheme was not a one time-off but long process that requires ample time.

Universal health coverage entails all people should access needed health care without experiencing catastrophic health spending. However, by December 2017, about half of the world’s population and big numbers of households suffered catastrophic health expenditure because of out of pocket payment for health care. Pooling is one of the core functions of health financing because it spreads financial risk across the population so that no individual carries the full burden of paying for health care. It is against this backcloth that the Kisiizi Hospital Health Insurance Scheme makes a contribution towards the achievement of universal health coverage. Lessons learned from Kisiizi Hospital Health Insurance Scheme can be applied to other similar settings with adjustments to meet the specific needs of different societies [34, 35].

## Conclusions

Solidarity in e-Societies formed a suitable foundation to begin Kisiizi Hospital Health Insurance scheme. Enforcement by e-Societies contributed significantly to enrolment and retention of membership in Kisiizi Hospital Health Insurance scheme. e-Societies also availed a loan facility to members and sometimes paid premiums for members using savings.

Community sensitization increased awareness and created demand for insurance while community participation was one of the strengths of Kisiizi Hospital Health Insurance scheme and promoted confidence and acceptability of Kisiizi Hospital Health Insurance scheme, helped to match premiums household resources, and partly addressed inequities.

Motivation to demand Kisiizi Hospital Health Insurance scheme cover was from fear of high costs of care, catastrophic health spending and e-Society disciplinary actions. Sensitization and sharing experiences between insured and non-insured made households appreciate benefits of subscribing to Kisiizi Hospital Health Insurance scheme and realised that they could generate funds for premiums and co-payments from sale of traditional non-cash crops and manual labour.

Availability of good quality health services was a matchless incentive and guaranteed insured members of value for their money. Kisiizi Hospital Health Insurance scheme facilitated access to these services which in turn attracted household members to enrol, subscribe and remain members.

Members of Kisiizi Hospital Health Insurance scheme enjoyed benefits such as; financial protection, access to good quality health services, and reduced sale of household assets. There was no more need to escape from the hospital bed before complete recovery as it used to happen in the past before Kisiizi Hospital Health Insurance scheme establishment.

However, a significant proportion of households were not insured. A bad past experience with insurance companies which never honoured their obligations made it difficult to mobilise community members to subscribe Kisiizi Hospital Health Insurance scheme. Others did not understand the importance of health insurance, how it operated and benefits. They did not perceive value for money in Kisiizi Hospital Health Insurance scheme and instead argued they could invest the money in business and pay out-of-pocket for health care from interest.

Kisiizi hospital achieved her objectives to; improve access to health services, and provide a stable source of funding and reduce burden of bad debts. Key findings of this study show that Kisiizi Hospital Health Insurance scheme met the cost of care for members and had a net balance, no debt to the hospital and did not need donations. Kisiizi Hospital Health Insurance scheme was self-reliant and sustainable in the long run.

### Lessons learned and policy implications

There were several success stories to learn from the development process of Kisiizi Hospital Health Insurance scheme that could useful for policy and decision makers. These were as follows;

- Kisiizi Hospital Health Insurance scheme tapped into cultural solidarity in e-Societies which provided a concrete foundation for it to begin, and enforced enrolment and retention of her members in the scheme. Most ethnic groups have cultures which could be exploited for a similar purpose.
- Availability of good quality health care was paramount and mandatory to generate confidence, demystify public scepticism and assure value for money. This provided incentives for enrolment, retention, and in Kisiizi Hospital Health Insurance scheme coverage.
- Relentless community sensitization increased awareness and created demand for Kisiizi Hospital Health Insurance scheme. Community participation in Kisiizi Hospital Health Insurance scheme contributed to making premiums resonate with household resources and expectations of the beneficiaries.
- Development of a health insurance scheme requires ample time and was not a one time-off process. It took Kisiizi Hospital Health Insurance Scheme 21 years and positive progress has been registered. Some of the established health insurance schemes took over 60 years to reach their current state. Resilience is success factor in the development of health insurance schemes.
- Community based health insurance schemes similar to Kisiizi Hospital Health Insurance scheme may not be replicated directly in other areas with different cultural complexities. They could be introduced and operated as savings and credit schemes with government capitation, and households make contribution using the local traditions as in the case of e-Societies. However, the net balance should be reserved by scheme and not surrendered to the health provider as it occurs in the case of Kisiizi Hospital Health Insurance scheme.
- There was potential for government to capitalise similar schemes and operate them as credit and savings schemes. Decreasing membership dues as savings increase, and enable the indigent to join the schemes and enjoy the benefits.
- There were areas for future research to understand possibility for portability of benefits within an effective referral system, and implementation of similar insurance schemes using Kisiizi Hospital Health Insurance scheme experience in different ethnic groups.

## Additional files

Additional file 1: Qualitative data from key informants. Key informant interview guide facilitated collection of data from three health providers and three chairpersons of e-societies. These were key actors in the Kisiizi Hospital Insurance Scheme with firsthand knowledge about it and its operations, and could provide information that was very useful in this study. (DOCX 15 kb)

Additional file 2: Qualitative data from discussions. Group discussion guide facilitated collection of data from a group of twenty community representatives (chairpersons of e-societies) and twenty four selected insured members from the community in three focus group discussions of eight members. This enabled researchers to generate valuable data and insights on Kisiizi Hospital Insurance Scheme from memories, ideas, experiences, perceptions, opinions, and attitudes of stakeholders in an interactive group setting with freedom to talk among group members. (DOCX 16 kb)

## Abbreviations

e-Society: Engozi Society
US$: United States of America Dollar
WHO: World Health Organization
