# Supplementary material for: Development of the Kisiizi hospital health insurance scheme: lessons learned and implications for universal health coverage
Source: BMC Health Serv Res. 2018 Jun 15;18:455. doi: 10.1186/s12913-018-3266-8 (PMC6003105; doi:10.1186/s12913-018-3266-8)
Supplement: Supplementary file 1 — Qualitative data from key informants. Key informant interview guide facilitated collection of data from three health providers and three chairpersons of e-societies. These were key actors in the Kisiizi Hospital Insurance Scheme with firsthand knowledge about it and its operations, and could provide information that was very useful in this study. (DOCX 15 kb) [file 12913_2018_3266_MOESM1_ESM.docx]

**ANNEX 1**

Key Informant Interview Guide

| Name of Key Informant |  |
| --- | --- |
| Position/title |  |
| Gender |  |
| Name of health facility |  |
| Years of work experience |  |
| Telephone |  |
| Date of interview |  |

**Note:** It will start with introduction of the research team and research project to the key informant. Each key informant is required to sign a consent form before giving any information.

**Researcher**

I would like us to discuss issues concerning the Kisiizi hospital health insurance scheme and I believe you are informed about it since its conception through implementation to date (please correct me where I make wrong assumptions).

1. Tell me a story about how it started to date and what have been your roles in the process (probe for influential factors for and against the scheme)

2. How was the idea of health insurance introduced to households and how was the idea received by the households?

3. From the households’ perspective, do they understand the concept of health insurance? What are the perceived problems and benefits?

4. Tell about the management of Kisiizi hospital health insurance scheme (probe: explain who are the key actors and how insured-households are handled at the point of health services consumption?).

5. Are the health services desired by patients available at Kisiizi hospital? (Probe: what happens when services needed are not available? If referred, who meets the costs?).

6. Let us talk about the health services at the Kisiizi hospital (Probe: quality, variety, health service packages, accessibility and utilization).

7. What are the sources of household income in the schemes’ or hospital’s catchment area?

8. Explain how membership into the scheme has grown over the life of the scheme.

9. Explain the enabling or facilitating factors and inhibitors that have influenced Kisiizi hospital health insurance scheme growth.

10. How can health insurance be promoted in your catchment area to ensure that all households subscribe to the Kisiizi hospital health insurance scheme?

11. Is there any important information that I have missed you can tell me?

Thank you very much for the information and effort given to me but please bear with me if I find missing information and come back to fill the gaps.

**END**
