# Supplementary material for: Development of the Kisiizi hospital health insurance scheme: lessons learned and implications for universal health coverage
Source: BMC Health Serv Res. 2018 Jun 15;18:455. doi: 10.1186/s12913-018-3266-8 (PMC6003105; doi:10.1186/s12913-018-3266-8)
Supplement: Supplementary file 2 — Qualitative data from discussions. Group discussion guide facilitated collection of data from a group of twenty community representatives (chairpersons of e-societies) and twenty four selected insured members from the community in three focus group discussions of eight members. This enabled researchers to generate valuable data and insights on Kisiizi Hospital Insurance Scheme from memories, ideas, experiences, perceptions, opinions, and attitudes of stakeholders in an interactive group setting with freedom to talk among group members. (DOCX 16 kb) [file 12913_2018_3266_MOESM2_ESM.docx]

**ANNEX 2**

Discussion Guide (household heads and representatives of societies)

| Name of group |  |
| --- | --- |
| Responsibility |  |
| Gender |  |
| Location |  |
| Duration of discussion |  |
| Date of interview |  |

Note: It will start with introduction of the research team and research project to the key informant. Each respondent is required to sign a consent form before giving any information and recording can take place.

**Moderator**

Moderator requests participants to introduce themselves and relation to the Kisiizi Hospital Health Insurance scheme.

I would like us to discuss issues concerning the Kisiizi hospital health insurance scheme and I believe you are informed about it since its conception through implementation to date (please correct me where I may make wrong assumptions).

1. Explain how you were selected to participate and your roles in the Kisiizi hospital health insurance scheme?

2. How was the idea of health insurance introduced to you, households and how was it received?

3. Do you and households understand the concept of health insurance? (Probe: arguments for and against the scheme, perceived problems and benefits).

4. Let us discuss the management of Kisiizi hospital health insurance scheme (probe: explain who are the key actors, and how insured-households are handled at the point of health services consumption and scheme funds?).

5. Are all health services desired by patients available at Kisiizi hospital? (Probe: what happens to the patient when services are not available? If the patient is referred, who meets the costs involved in the referral system? Does the scheme reimburse costs e.g. emergency costs of health care received at other health facilities).

6. Let us discuss the health services available at the Kisiizi hospital (Probe: quality, variety, health service packages, accessibility, waiting time and utilization).

7. What are the sources of household income in the schemes’ or hospital’s catchment area?

8. Explain how membership into the scheme has grown since it began and why some households in your villages have not subscribed to the Kisiizi hospital health insurance scheme.

9. Explain the factors that influence subscription, drop out and inability to be member of the Kisiizi hospital health insurance scheme.

10. Discuss how health insurance can be promoted to ensure that all households subscribe to the Kisiizi hospital health insurance scheme?

11. Is there any important information that I have missed and you can tell me?

Thank you very much for participating in this discussion, the valuable information given to us, and your time. Please bear with me if I find missing information and come back to fill the gaps.

**END**
